# Vascular Manifestations of COVID-19 – Thromboembolism and Microvascular Dysfunction

**DOI:** 10.3389/fcvm.2020.598400

**Published:** 2020-10-26

**Authors:** Kirsty A. Roberts, Liam Colley, Thomas A. Agbaedeng, Georgina M. Ellison-Hughes, Mark D. Ross

**Affiliations:** ^1^Research Institute for Sport and Exercise Sciences, Liverpool John Moores University, Liverpool, United Kingdom; ^2^School of Sport, Health & Exercise Science, Bangor University, Bangor, United Kingdom; ^3^Centre for Heart Rhythm Disorders, School of Medicine, The University of Adelaide, Adelaide, SA, Australia; ^4^Centre for Human and Physiological Sciences, Faculty of Life Sciences & Medicine, School of Basic and Medical Biosciences, King's College London, London, United Kingdom; ^5^School of Applied Sciences, Edinburgh Napier University, Edinburgh, United Kingdom

**Keywords:** COVID-19, endothelium, pericyte, coronavirus, thromboembolism

## Abstract

The coronavirus pandemic has reportedly infected over 31.5 million individuals and caused over 970,000 deaths worldwide (as of 22nd Sept 2020). This novel coronavirus, officially named severe acute respiratory syndrome coronavirus 2 (SARS-CoV-2), although primarily causes significant respiratory distress, can have significant deleterious effects on the cardiovascular system. Severe cases of the virus frequently result in respiratory distress requiring mechanical ventilation, often seen, but not confined to, individuals with pre-existing hypertension and cardiovascular disease, potentially due to the fact that the virus can enter the circulation via the lung alveoli. Here the virus can directly infect vascular tissues, via TMPRSS2 spike glycoprotein priming, thereby facilitating ACE-2-mediated viral entry. Clinical manifestations, such as vasculitis, have been detected in a number of vascular beds (e.g., lungs, heart, and kidneys), with thromboembolism being observed in patients suffering from severe coronavirus disease (COVID-19), suggesting the virus perturbs the vasculature, leading to vascular dysfunction. Activation of endothelial cells via the immune-mediated inflammatory response and viral infection of either endothelial cells or cells involved in endothelial homeostasis, are some of the multifaceted mechanisms potentially involved in the pathogenesis of vascular dysfunction within COVID-19 patients. In this review, we examine the evidence of vascular manifestations of SARS-CoV-2, the potential mechanism(s) of entry into vascular tissue and the contribution of endothelial cell dysfunction and cellular crosstalk in this vascular tropism of SARS-CoV-2. Moreover, we discuss the current evidence on hypercoagulability and how it relates to increased microvascular thromboembolic complications in COVID-19.

## Introduction

In January 2020, the Center for Disease Control recognized a new coronavirus, named severe acute respiratory syndrome coronavirus 2 (SARS-CoV-2), which is believed to have originated from the Wuhan city in Hubei province, China. As of the 22nd September 2020, over 31.5 million people worldwide have been infected, with currently over 970,000 deaths recorded (1). According to the World Health Organization (WHO) the total case fatality rate (CFR) is 3.1%, but this varies significantly depending on geographical location. For example, the USA have a CFR of 2.9% (6,740,464 cases), whereas the United Kingdom and Italy have significantly higher CFRs of 10.6% (394,261 cases) and 12.0% (298,156 cases), respectively (1). The SARS-CoV-2 infection gives rise to COVID-19 disease, which typically results in fever, respiratory distress (shortness of breath and cough) (2–4), and subsequent respiratory failure. Symptoms often arise between 2 and 14 days after infection (5), and the risk of mortality due to COVID-19 appears greater in older individuals (6), and in individuals with comorbidities, such as hypertension (7), coronary artery disease (CAD), and diabetes mellitus.

Despite patients reporting with symptoms relating to fever and respiratory distress, there is growing evidence for the involvement of the cardiovascular system. Patients often exhibit elevated cardiac biomarkers such as cardiac troponin I/T (hs-cTnI/hs-cTnT) (3, 4, 6, 8–11) and N-terminal pro-B-type natriuretic peptide (NT-proBNP) levels (8, 12), which suggest myocardial damage and ventricular/atrial dysfunction. However, the impact of COVID-19 on the vasculature is largely unknown, but there are case reports of viral infection of the endothelium (13), as well as elevated markers of coagulation, such as D-dimer in COVID-19 patients (14), which itself may indicate a significant risk of pulmonary thromboembolism (PTE) in patients.

The focus of this review is to detail the effects of SARS-CoV-2 and COVID-19 disease on the vasculature, whilst discussing the potential direct and indirect mechanisms which lead to endothelial damage and dysfunction. Moreover, we also discuss the pathogenesis of COVID-19 associated thromboembolism and its consequences upon the cardiovascular system and COVID-19 disease progression.

## Epidemiology of COVID-19 and Cardiovascular Risk

Patient cohort studies show that there is a large prevalence of patients with COVID-19 who have comorbidities, such as hypertension (17–57% of all patients) and cardiovascular disease (CVD) (11–21% of all patients) (3, 15–17). Patients with hypertension or CAD are not only at greater risk of infection, and admission to hospital, but having one or more of these comorbidities also appears to increase the risk of progression of the disease (15). In a Chinese cohort, it was observed that in COVID-19 patients, 30% of them had hypertension (14). In the non-survivors, the incidence of hypertension was greater than that of survivors (48 vs. 23% of patients), and this was even more pronounced for incident coronary heart disease (24 vs. 1% of patients) (14). Hypertension and pre-existing CVD were also more common comorbidities in patients requiring admission to the intensive care unit (ICU) (18).

The initial evidence of the cardiovascular impact of COVID-19 was provided in cross-sectional cohort studies which observed significantly elevated hs-cTnI and hs-cTnT levels, suggestive of myocardial injury in these patients (14, 18, 19). High levels of these cardiac biomarkers are related to worse prognosis of the disease (19, 20), with a number of studies demonstrating a higher risk of admission to ICU (10), requirement for mechanical ventilation (12), and incidence of arrhythmias and death from COVID-19 (3, 4, 10, 12, 19) in those with elevated circulating hs-cTnI or hs-cTnT levels. Moreover, the mortality risk associated with elevated hs-TnI/T was greater than that observed for advanced age, pre-existing diabetes, respiratory disorders, and CAD (10, 12). The elevations in hs-TnI/T are also associated with elevated levels of NT-ProBNP and C-reactive protein (CRP), suggesting the myocardial injury observed in COVID-19 patients may be linked with ventricular dysfunction and inflammation (12). There are several potential reasons for the elevated cardiac injury observed in COVID-19 patients with worsening outcomes. These include direct viral infection of the myocardium, the use of anti-viral medications (18), the side-effects of the COVID-19 associated cytokine storm (21), or likely a combination of the three. Viral entry is likely, as SARS-CoV-2 is known to enter human cells via binding of the transmembrane protein, the angiotensin-converting enzyme 2 (ACE2) receptor, which is highly expressed in both the lungs and the heart (22). In fact, due to this mechanism of entry, there has been debate on the use and potential benefit of the use of ACE inhibitors in patients with cardiac injury and/or hypertension (23), with the American Heart Association, The Heart Failure Society of America, and the American College of Cardiology publishing a joint consensus statement for the treatment of COVID-19 patients with ACE inhibitors (24).

Cardiovascular events, such as incidences of acute coronary syndrome (ACS) or acute myocardial infarction (AMI) in COVID-19 patients have been demonstrated (25), indicating that the impact of COVID-19 on the cardiovascular system leads to cardiovascular-related mortality. The root causes of COVID-19 ACS/AMI remain unknown, but could be due to the elevated myocardial demand as a result of the infection, akin to type 2 MI, cytokine-induced atherosclerotic plaque instability and rupture, or non-plaque thrombosis (25–27). Although, as documented, there is a clear impact of the virus on the myocardium, either directly or indirectly; however, the potential role of the vasculature in COVID-19 associated cardiovascular complications has been relatively overlooked, and may be prognostically important in these patients. In fact, in a recent study by Chen et al. (28) using a single cell atlas of the human myocardium showed that ACE2 is expressed on pericytes in the heart (28), suggesting that viral infection of pericytes, which surround the endothelial lining of blood vessels, could lead to microvascular inflammation in the heart tissue, resulting in non-obstructive MI. Therefore, the following sections will investigate the impact of COVID-19 on vascular tissues, specifically endothelial cells and pericytes, and the subsequent involvement of these tissues on thrombotic risk in COVID-19.

## COVID-19 and Endothelial Cell Dysfunction

Initial SARS-CoV-2 infection occurs within the lung epithelia, whereby serine proteases, most notably transmembrane protease serine 2 (TMPRSS2), cathepsin B, and cathepsin L1, prime the SARS-CoV-2 spike glycoprotein, which is followed by ACE2-mediated viral entry (29). Infection of lung alveoli allows SARS-CoV-2 to enter the systemic circulation, subsequently predisposing multiple organs to potential infection. Co-expression of both key serine proteases and ACE2 is required for successful infection of cells by SARS-CoV-2 (29). Multiple organs contain cells which co-express ACE2 and these serine proteases, including the lungs, heart, kidneys, liver, and the vasculature (30–32).

Microvascular dysfunction and the role of the vascular endothelium is increasingly implicated in the acute respiratory distress syndrome (ARDS) and systemic impact of SARS-CoV-2 infection. Endothelial cells protect the cardiovascular system and are crucial in regulating vascular homeostasis, preventing coagulation, controlling blood flow, and regulating oxidative stress and inflammatory reactions (33, 34). There is growing evidence of a vascular involvement in the pathogenesis of severe COVID-19, with imaging studies revealing perfusion abnormalities within the brains of patients with COVID-19 presenting with neurological issues (35), in addition to perfusion abnormalities within the lungs of COVID-19 pneumonia patients (36). Moreover, cross-sectional studies have reported a high incidence of coagulopathies, characterized by elevated D-dimer and fibrinogen concentrations, which lead to thrombotic events and are associated with poor outcomes (37, 38), thus demonstrating the potential involvement of endothelial cells in the pathophysiological consequences of COVID-19.

### Endothelial Cell Involvement in COVID-19

Involvement of endothelial cells in the pathophysiology of COVID-19 goes beyond coagulation derangements, with SARS-CoV-2 being shown to directly infect engineered human blood vessel organoids and human kidney organoids *in vitro* (39). This has been confirmed, *in vivo*, by histological studies demonstrating viral infiltration into endothelial cells, with Varga et al. (13) reporting endothelial cell involvement across multiple organs (e.g., lungs, heart, intestines, kidneys, and liver) in three patients; two of whom died (multisystem organ failure; myocardial infarction, and subsequent cardiac arrest, respectively) and one survived. Viral infection of endothelial cells was observed in a transplanted kidney of one patient with evidence of endothelial cell inflammation (endothelialitis) within cardiac, small bowel, lung, and liver tissue of two patients. Furthermore, one other patient demonstrated endothelialitis of the submucosal vessels within the small intestine, which was accompanied by a reduced left ventricular ejection fraction. These findings demonstrate direct viral infection of endothelial cells and endothelialitis within multiple tissue beds in patients with COVID-19.

Although limited by a small sample size, the findings of Varga et al. (13) are supported by Ackermann et al. (40), who reported severe endothelial injury, viral infection, and disrupted cell membranes in seven lungs obtained post-mortem from individuals who died from COVID-19. When compared to seven lungs from individuals who died from influenza, microthrombi were nine times as prevalent in the lungs from the COVID-19 individuals. Furthermore, widespread microthrombi was accompanied by microangiopathy and occlusion of alveolar capillaries (40), which is in line with other studies (41), and can predispose organs to microinfarcts (42). An unexpected finding was the observation of intussusceptive angiogenesis, in which the degree was associated with the duration of hospitalization (40). Intussusceptive angiogenesis is the formation of new vessels, via non-sprouting angiogenesis, and is constructed of an endothelial-lined “pillar” spanning the vessel lumen, which significantly alters the microcirculation (43). Cytoplasmic vacuolisation and cell detachment in pulmonary arteries (44), in addition to pulmonary capillary injury featuring neutrophil infiltration and fibrin deposition (41, 45) has also been reported, further demonstrating local endothelial cell perturbations within lung tissue. Moreover, renal post-mortem histopathological analysis by Su et al. (46) found endothelial cell swelling with foamy degeneration in 19% of patients, with 12% demonstrating a few areas of segmental fibrin thrombus in glomerular capillary loops that is associated with severe endothelial injury.

**Figure 1:**
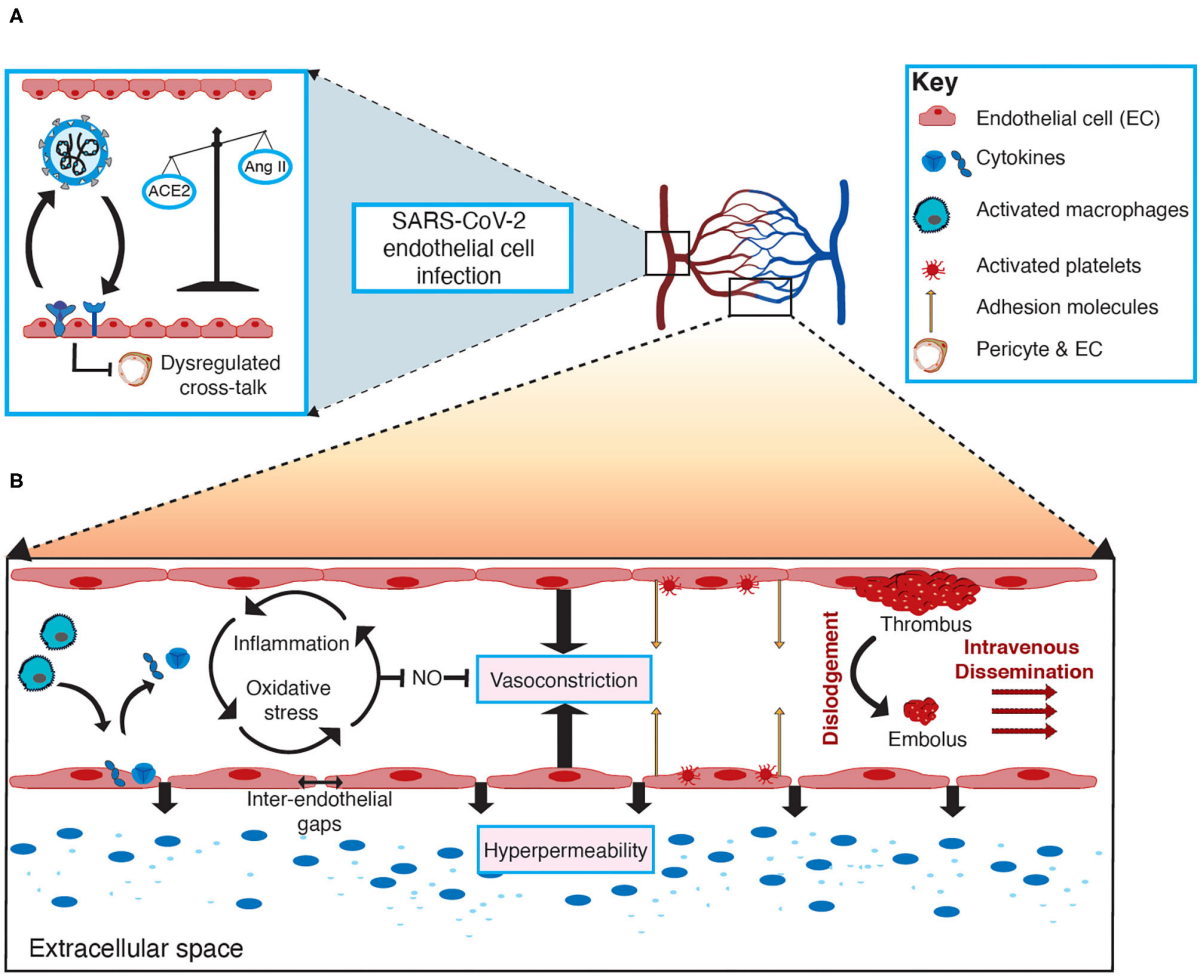
The role of endothelial cells and mechanisms of endothelial cell dysfunction in COVID-19. **(A)** SARS-CoV-2 infects endothelial cells through angiotensin-converting enzyme 2 (ACE2) mediated viral entry, facilitated by TMPRSS2 priming the SARS-CoV-2 spike glycoprotein. Infection of endothelial cells may result in a downregulation of ACE2, promoting an imbalance between ACE2 and angiotensin II (AngII) levels, in favor of AngII. Moreover, infection of either endothelial cells or pericytes will perturb the crosstalk between these two cells, thus contributing to endothelial cell dysfunction. **(B)** In severe cases of COVID-19, activated macrophages release various cytokines (e.g., soluble interleukin 2-receptor [IL-2R], interleukin-6 [IL-6] and tumor necrosis factors [TNFs]), which are attributed to the exaggerated immune-mediated cytokine storm and can result in vascular inflammation (endothelialitis) as a result of increased adhesion molecule expression on endothelial cells and inter-endothelial gaps, thus promoting vascular hyperpermeability. Activated endothelial cells can contribute to the cytokine storm by releasing various cytokines in response to damage and dysfunction, contributing to a vicious cycle of inflammation and oxidative stress that inhibits the release of vasoactive factors (e.g., nitric oxide [NO]), thus favoring vasoconstriction and further contributing to vascular permeability. Abnormal activation of platelets and endothelial cells is the key process leading to thrombosis, which represents the role of endothelial cell dysfunction in the pathogenesis of thromboembolism in COVID-19 patients. Subsequently, the dislodgement of thrombotic clots creates a mobile embolus that disseminates intravenously, thereby leading to thromboembolic complications in COVID-19.

Considering endothelial dysfunction leads to impaired systemic microvascular function, it seems likely that involvement of the vascular system's first line of defense (endothelial cells) precipitates and propagates the systemic damage observed in severe cases of COVID-19, through altered vascular integrity, vascular inflammation, and via disruption of coagulation and inflammatory pathways (13, 33). The mechanisms for this have not yet been fully elucidated and are varied due to the heterogenic nature in which the virus affects individuals. Cardiometabolic comorbidities associated with poorer prognosis in COVID-19 patients have a strong association with pre-existing endothelial dysfunction (i.e., hypertension and CAD) (47, 48). It is therefore evident that understanding the role of endothelial cells in SARS-CoV-2 infection is crucial to identifying potential therapeutic strategies to combat the virus and improve patient outcomes. The role of endothelial cells and potential mechanisms of endothelial cell dysfunction in COVID-19 are depicted in Figure 1.

### Potential Mechanisms of Endothelial Dysfunction in COVID-19

#### Angiotensin-Converting Enzyme 2 (ACE2)

ACE2 is an endogenous negative regulator of the renin-angiotensin system (RAS) and has been identified as the key receptor facilitating viral entry of SARS-COV-2 (49, 50), along with key serine proteases to prime the spike glycoprotein of the virus, most notably TMPRSS2 (29), which is expressed by endothelial cells (30). ACE2 is widely expressed in cells throughout the body, from the respiratory tree to the vascular system, heart, kidneys, liver, gut, central nervous system, and retina, and is recognized as eliciting protective effects, particularly against CVD (49). The expression of ACE2 in many organs allows relatively easy transport of the virus throughout the body (51). Consequently, interference of the physiological processes associated with ACE2 by viral entry of SARS-CoV-2 is likely to explain the multi-organ dysfunction pertaining to endothelial cells that is seen in severe cases of COVID-19.

A downregulation in the expression of ACE2, as a result of viral entry into cells, disrupts the regulation balance between angiotensin II (Ang II) and ACE2, indirectly affecting the vasculature. This imbalance facilitates an elevation in the expression of Ang II, subsequently promoting an atherogenic state across the cardiovascular system, especially inflammation and oxidative stress, whilst also elevating blood pressure by stimulating an increase in sympathetic nervous system activity (52). This is supported by studies reporting marked elevations in plasma AngII concentrations in patients with COVID-19 (53) and also being linked to disease severity in patients infected with novel influenza A (54). This pathophysiological increase in Ang II and without the modulator and protective effects of Ang 1-7, results in downstream elevation of plasminogen activator inhibitor-1 (PAI-1) from endothelial cells, further accelerating vascular inflammation and the facilitation of the coagulation cascade (42), thus resulting in endothelial damage (55). Elevated PAI-1 is a hallmark of endothelial dysfunction, promoting increases in circulating endothelial microvesicles, resulting from endothelial shedding via activated cells, which pose a risk of thromboembolic events (56, 57).

Some have argued that following cell entry of SARS-CoV-2, down-regulation of ACE2 receptors may result in an indirect activation of the kallikrein-bradykinin pathway, thereby promoting an increase in vascular permeability and thus leading to oedema and microcirculatory dysfunction (33, 58, 59). It has been suggested that kinin inhibition may be a potential therapeutic approach to reducing vascular leakage into the lung, and therefore, oedema (60). Kinin inhibition may, therefore, promote endothelial repair through reducing vascular permeability, although whether this is an effective therapeutic approach is yet to be confirmed within the literature. In contrast to this, consistent reports of hypokalaemia in patients with severe COVID-19 (61, 62) suggest an increase in aldosterone, via elevations in Ang II, resulting in an increase in ACE, which acts to metabolize bradykinin (63). Therefore, the role of bradykinin in the pathogenesis of microvascular dysfunction in COVID-19 is questionable and more likely a result of the effects of Ang II, stemming from a downregulation of ACE2 after viral entry into cells. Moreover, given that hypokalaemia is associated with ventricular arrhythmias that are commonly observed in COVID-19 (18), it is plausible that this is a contributing mechanism to both endothelial dysfunction and arrhythmogenesis.

#### The Cytokine Storm

The mechanisms involved in the pathogenesis of microvascular dysfunction in COVID-19 patients, although not yet fully understood, are likely not solely attributed to direct viral infection of endothelial cells. Endocytosis or membrane fusion of SARS-CoV-2 to cells either leads to cell damage or apoptosis which activates the immune response and the release of various cytokines promoting an exaggerated inflammatory environment (42). Moreover, endothelial cells regulate local and systemic inflammatory reactions and immune responses (33) and activation of these cells via the exaggerated immune-mediated inflammatory response of SARS-CoV-2 may present an indirect mechanism of endothelial damage and dysfunction among the COVID-19 patient population. Endothelial cells produce various cytokines and chemokines and have been identified as central regulators of an exaggerated systemic inflammatory response, or “cytokine storm” (64), a common feature of severe SARS-CoV-2 infection (65).

More severe cases of COVID-19 are associated with progressive lung damage which has, in part, been attributed to this cytokine storm (65–67), leading to a loss of vascular barrier integrity and likely promoting pulmonary oedema, thereby causing endothelialitis, and activation of coagulation pathways. Cross-sectional studies have consistently demonstrated marked elevations in pro-inflammatory markers, such as soluble interleukin-2 receptor (IL-2R), interleukin-6 (IL-6), CRP, and tumor necrosis factors (TNF) (6, 12, 68). This marked elevation in pro-inflammatory markers has been linked with mortality and promotes inter-endothelial gaps and thus vascular hyperpermeability (69, 70), along with exacerbating oxidative stress. IL-6 in particular is associated with increased vascular permeability, a hallmark of the inflammatory response (71, 72), and IL-6 levels are directly correlated with the severity and mortality of COVID-19 (14, 73, 74). Moreover, IL-6, along with other cytokines released from activated macrophages, such as IL-1β, activate endothelial cells via elevations in adhesion molecules (42) leading to a myriad of vascular disturbances including leukocyte tethering to the vascular bed, platelet aggregation and coagulation derangements.

#### Oxidative Stress

An overproduction of reactive oxygen species (ROS) in infected cells is a key factor in viral replication of respiratory viruses and subsequent tissue damage (75). Following viral infection, endothelial activation and regulation of adhesion molecules leads to neutrophil activation, which results in the production of a plethora of histotoxic mediators including ROS (59). This has implications for the onset and progression of the cytokine storm since, as described above, endothelial cells are key orchestrators of cytokine overload. The ensuing oxidative stress, defined as a systemic imbalance between ROS (or free radicals) and antioxidants, causes an increased expression of prothrombotic and cell-surface adhesion molecules (76). Oxidative stress may therefore be linked to the pathogenesis and severity of COVID-19 infections (77) and peri-endothelial ROS production in COVID-19 may, therefore, contribute to the multi-organ failure associated with severe disease, which seems likely given that it has previously been demonstrated in the pathogenesis of other viral infections, such as SARS-CoV and influenza (78, 79), and ARDS (80). The elevation in ROS accumulation promotes oxidative stress and nuclear factor kappa B (NF-κB) signaling, with the potential for dysregulated antioxidant mechanisms, such as Nrf2 and antioxidant response element signaling, promoting the release of various endothelial genes, such as endothelin and adhesion molecules, thus favoring vasoconstriction and increased vascular permeability (81, 82).

The elevation in free radical production, potentially as a combined result of increased Ang II expression, pro-inflammatory responses, and a reduced capacity for free radical scavenging by impaired antioxidant signaling, impairs endothelial function. Elevated superoxide concentrations, promoted by the release of mitochondrial-derived ROS is a hallmark of oxidative stress, which facilitates the quenching of nitric oxide (NO) and the formation of the secondary free radical, peroxynitrite, in turn reducing NO bioavailability (83). Moreover, this process uncouples endothelial nitric oxide synthase, which further elevates superoxide production, contributing to the pro-oxidant environment of the vasculature. Such elevations in oxidative stress would promote antioxidant signaling, however, numerous respiratory viral infections, such as respiratory syncytial virus, human metapneumovirus, and influenza, have perturbed antioxidant defense mechanisms by inhibiting antioxidant enzyme induction (84). Interestingly, it has been proposed that Nrf2 activators could be a potential therapeutic strategy for inhibiting viral entry of SARS-CoV-2 (85), and may also pose a benefit to endothelial repair and functioning by the scavenging of free radicals, reducing oxidative stress, and inhibiting pro-inflammatory signaling.

#### Coagulation Cascade

Perturbations to the endothelium may result in vascular leakage and promote inflammation, but also predispose the vasculature to a pro-coagulant state. Indeed, a common manifestation in patients with COVID-19 is the presence of coagulation abnormalities and instances of thromboembolism, which has been associated with disease severity and a higher incidence of mortality (38), whilst also increasing the risk of MI and stroke. The endothelium plays an important role in the prevention of thromboembolic events by regulating the coagulation cascade, achieved, in part, via inhibition of various tissue factors by a Kunitz-type protease inhibitor, known as the tissue factor pathway inhibitor (TFPI) that resides on the endothelial cell surface (34). The transmembrane protein tissue factor is required for *in vivo* coagulation by the binding and activation of various tissue factors (i.e., activation of factor Xa) promoting prothrombin conversion to thrombin, and thus the conversion of fibrinogen to fibrin (34, 86), inhibiting TFPI and promoting clot formation. TFPI is predominantly bound to the microvasculature (87), however, it has been demonstrated to play a role in the regulation of arterial thrombosis in mice (86).

Marked coagulation derangements have been reported in a single-center cross-sectional study by Goshua et al. (88) who assessed markers of endothelial cell and platelet activation, namely circulating von Willebrand factor (vWF), soluble P-selectin and soluble thrombomodulin, in critically and non-critically ill COVID-19 patients. They observed that endotheliopathy is present in COVID-19 and is associated with increased mortality, with a suggestion that soluble thrombomodulin concentrations may predict mortality and clinical outcomes in COVID-19 patients. It was suggested that the coagulopathy observed in their data was distinctly separate from disseminated intravascular coagulation (DIC) and should be considered an endotheliopathy (88). The notion of a “COVID-19 coagulopathy” is supported by a number of other studies. DIC has been reported to be characteristic of COVID-19, however, its presentation is different to that regularly observed in sepsis-induced DIC. In sepsis-induced DIC, marked thrombocytopenia is observed with a mild elevation in D-dimer concentrations (89), which is in contrast to DIC observed in COVID-19 patients (90). This is supported by only 14.7% (22 of 150) of patients scoring positive on the “sepsis-induced coagulopathy score” (90). DIC has been linked with multi-organ system failure within the COVID-19 population (38, 91, 92), demonstrating a pro-coagulant state of the vasculature. Furthermore, mild thrombocytopenia can be found in 70 to 95% of patients with severe COVID-19, however, it has not been found to be an important predictor of outcome (21, 93). Therefore, the presence of coagulopathy within patients with COVID-19 should be considered as an endotheliopathy, rather than traditional DIC.

#### Cellular Cross-Talk: Endothelial Cells and Pericytes

Pericytes share a basement membrane with endothelial cells, which is formed, maintained, and remodeled successfully through cellular cross-talk between these two cells, demonstrating that pericytes and endothelial cells have an extensive linkage and are key for maintaining basement membrane, and thus vascular barrier integrity. This has been confirmed by cell-to-cell interaction analysis, demonstrating that endothelial cells are the main cross-talking cell with pericytes within cardiac tissue, with a predominant role of angiopoietin ligands (ANGPT1/2) and Tie receptor 2 (TIE2) maintaining endothelial cell stability and function in capillary vessels (28). A balance between ANGPTs and TIE2 is key for the maintenance of endothelial stability and vascular integrity (28, 94); therefore, it is possible that a breakdown of the cross-talk between pericytes and endothelial cells disrupts this balance and results in a compromised vasculature that is prone to a pro-inflammatory, pro-coagulant state. Whilst these findings were observed in normal heart tissue, this is supported by a pericyte-specific infection by SARS-CoV-2 in experimental (95) and human histological studies (96).

Whilst there is evidence of a direct viral infection of endothelial cells, some have argued that endothelial cell dysfunction is a result of pericyte infection. Cardot-Leccia et al. (96) reported wall thickening of the venules and alveolar capillaries in lung tissue of a deceased COVID-19 patient, accompanied by a marked decrease in pericytes, compared to normal lung parenchyma. Combined with the findings of He et al. (95) and the highly infectious potential of pericytes demonstrated by single cell RNA sequencing studies (28), these data seem to support a potential “pericyte hypothesis” as a mechanism for microvascular dysfunction in the pathogenesis of COVID-19. Moreover, infection and loss of pericytes would result in a dysregulation of the cross-talk between pericytes and endothelial cells, promoting capillary endothelial dysfunction, which would explain the wall thickening of venules and capillaries observed in the data from Cardot-Leccia et al. (96). Taken together, pericytes seem to have the potential as a highly infectious cell population for SARS-CoV-2 and may contribute to endothelial dysfunction by promoting an imbalance between ANGPT1/2 and TIE2, perturbing vascular barrier integrity and increasing vascular permeability. However, the notion that it is solely pericytes that are infected and induce endothelial dysfunction is unlikely considering the compelling histological data presented within the literature (13, 40).

## COVID-19 and the Coagulation Cascade - Risk of Thromboembolic Events

There is evidence to suggest increased risk of thrombotic complications and stroke (both are hereafter referred to as thromboembolism for simplicity) in COVID-19 (97). At the mechanistic level, both venous and arterial thrombosis have been attributed to activation of inflammation and hypoxia, platelet activation, endothelial dysfunction, and circulatory stasis. However, the impact of thromboembolic complications on the prognosis of COVID-19, clinical course of thromboembolic disorders in these patients, and the impact of prophylactic and therapeutic anticoagulation therapies in COVID-19 are not well-known.

### Epidemiological Burden of Thromboembolism in COVID-19

The prevalence of neurologic manifestations, including cerebrovascular diseases, was reported at 36.4% in an earlier retrospective case series from Wuhan, China (98). In patients presenting with confirmed or suspected COVID-19, thromboembolism is prevalent at 20.4% (99). In the same study, six of the patients with laboratory findings demonstrated elevated D-dimer levels (>7,000 mg/L) and 40% of the patients had pulmonary thromboembolism. Another series showed that 67% of thromboembolic complications are ischaemic in origin, while 33% are haemorrhagic (100). In the pediatric population, thromboembolic complications are not common. For instance, elevation of D-dimer was not found in children with SARS-CoV-2 compared to other inflammatory multisystem syndromes (101), and no thromboembolic event was found in children and adolescents in a large, multicentre European cohort (102).

In addition to a prior history of stroke, patients with COVID-19 develop incident thromboembolism. The incidence rates of acute thromboembolic complications are reported between 5 and 32.5% in retrospective cohorts (103, 104). Underlying cardiovascular risk factors, including diabetes, hypertension, and a history of CVD, are implicated as univariate correlates (103). D-dimer levels at hospital admission are also significantly correlated with incident thromboembolism, with a negative predictive value of more than 90% (104). In a prospective cohort of 150 French COVID-19 patients vs. a historic cohort of 233 non-COVID-19 controls, COVID-19 ARDS independently predicted thromboembolic complications and pulmonary thromboembolism even after propensity score matching (90).

The comorbid nature of thromboembolic lesions in patients with COVID-19 underscores some underlying predisposition to SARS-CoV-2 infection. Indeed, thromboembolic complications have been associated with depressed immune function and increased post-stroke infections. Infection rates ranging from 18.7 to 43.7% have been reported in patients with intracerebral hemorrhage (105, 106), with respiratory infections predicting almost six-fold higher risk of future thromboembolism (106). A 1-unit increment in National Institutes of Health Stroke Scale (NIHSS) was associated with 23% increased risk of COVID-19 positivity. Interestingly, in a retrospective multicentre study of stroke patients (107), 28% were later diagnosed with COVID-19. However, the true burden of thromboembolism COVID-19 remains unknown and will, hopefully, be answered by larger prospective studies.

### Impact of Thromboembolic Complications on COVID-19 Prognosis

The presence of underlying or incident thromboembolic complications is associated with poor prognosis of COVID-19. A history of thromboembolism is reported in 2.3 to 22% of severe cases compared to 0 to 6% in non-severe cases (108). Patients with prior neurologic thromboembolic complications are shown to have a 2.5-fold increased risk of COVID-19 severity (108) and D-dimer is often elevated above reference range in hospitalized cases (17). These patients are usually older, have a higher number of comorbidities, have a higher prevalence of ARDS, and are more likely to be non-invasively ventilated (109). Data also shows that patients with more severe COVID-19 have higher incidence rates of thromboembolic complications. For instance, 31% of patients admitted to the ICU developed thromboembolic complications during follow-up in one Dutch study (110). Yearly increment in age and prior coagulopathy, defined as prothrombin time >3 s or activated partial thromboplastin time (aPPT) >5 s, are shown as independent predictors of incident thromboembolic complications in severe COVID-19 (110). Diagnosis of pulmonary thromboembolism in ICU patients with COVID-19 is more common (at 21%) compared to 7% admitted due to influenza or 6% for all ICU patients (111).

Additionally, the association between a history of thromboembolic complications and mortality has been analyzed in COVID-19 patients. The burden of underlying coagulopathy was reported in 50% of non-survivors in the Wuhan cases (14), with a D-dimer >1,000 ng/mL (reference range ≤250 ng/mL) shown to be an independent predictor of 18-fold greater risk of in-hospital mortality (14). A multicentre cohort from the US showed that the coagulation component of the SOFA score is associated with 64% greater odds of 28-day in-hospital death in a multivariable adjusted model (112). These observations are further supported by the results of a meta-analysis (113), which show a 2.4-fold elevated risk of mortality in COVID-19 patients with cerebrovascular disease, defined as stroke and brain infarction. Overall, these data highlight the risk, and subsequent poor prognosis of thromboembolism in COVID-19.

### Coagulation Cascades and the Mechanisms of Thrombosis in COVID-19

**Figure 2:**
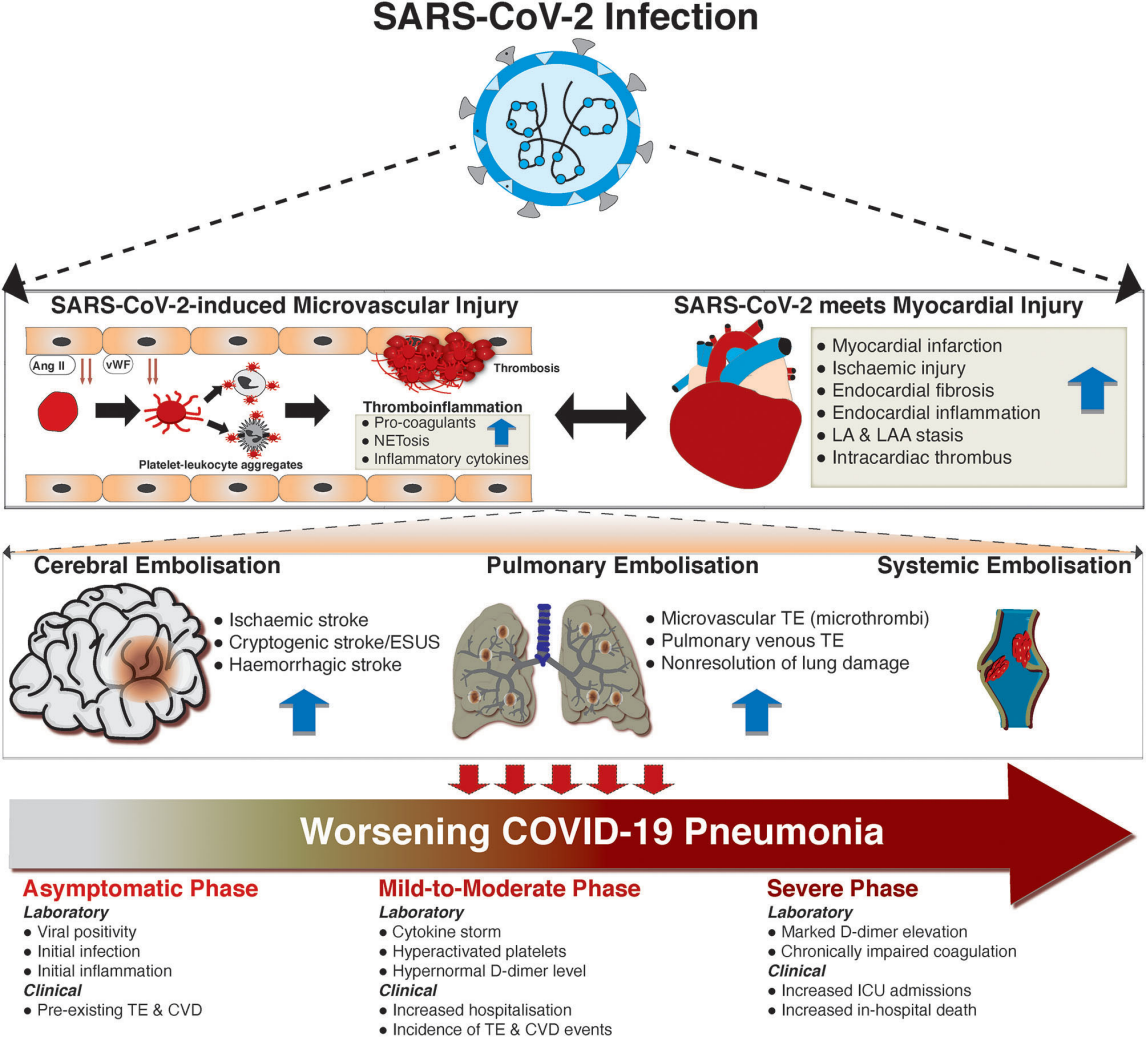
The development and consequences of thromboembolism in COVID-19. The thromboembolic implications of SARS-CoV-2 are best conceptualized in three key stages. First, lung infection of SARS-CoV-2 can spill over, with a consequent cardiovascular tropism of the virus. Within the vascular beds, the increased level of Ang II, which occurs due to SARS-CoV-2 mediated depletion of ACE2, could drive the dysfunction of endothelial cells. This, and other independent pathways (i.e., direct infection of endothelial cells), could lead to the release of von Willebrand factors (vWF), which can activate circulating platelets via adhesive glycoprotein receptors (i.e., gpIb). Activated platelets form aggregates with monocytes and neutrophils, leading to enhanced production of pro-coagulants, inflammatory cytokines, and neutrophil-extracellular traps (NETosis). Within the heart, SARS-CoV-2 infection can directly and indirectly (via cytokine storm) lead to myocardial ischaemia, myocardial infarction, endocardial dysfunction (via inflammation and subsequent fibrosis), and blood stasis in the left atrial atrium (LA) and left atrial appendage (LAA). These can, in turn, lead to intracardiac thrombus. Moreover, thromboinflammation within the vascular beds can drive myocardial injury and vice versa. In the second stage, the dislodgement of thrombus creates mobile embolus, which can be carried to the brain (causing stroke), pulmonary vasculature (causing pulmonary thromboembolism [TE]), or systemically (causing venous thrombosis). Importantly, the presence of thromboembolic complications can lead to progressive COVID-19 disease (in the third conceptual stage). The presence of underlying cardiovascular disease (CVD; i.e., TE) could predispose individuals to SARS-CoV-2 infection via inflammatory derangement. Coexistence of SARS-CoV-2 infection and TE can lead to dysregulated inflammation and coagulation disorders, manifesting with high symptom burden and hospitalization, and increased *de novo* incidence of TE and other CVDs. Consequently, TE and CVDs predispose COVID-19 patients to worse outcomes, including prolonged intensive care unit (ICU) stay and in-hospital mortality.

While significant associations have been noted for thromboembolism and SARS-CoV-2 infection and worsening of COVID-19, a causal relationship is not well-defined. However, there are data to suggest some mechanistic underpinnings (Figure 2). Laboratory investigations have demonstrated significant elevations of markers of coagulation cascades, such as D-dimer, aPPT, fibrinogen, and factor VIII. D-dimer ≥2,600 ng/mL and failure of clot lysis at 30 min on thromboelastography predicted future thromboembolic events in ICU patients with c-statistic of 0.78 and 0.74, respectively (114). This highlights the fact that shutdown of fibrinolysis occurs in COVID-19. In addition to coagulation markers, endothelial dysfunction may underlie the increased risk of thromboembolism in COVID-19 as both vWF activity and vWF antigen are increased in COVID-19 ARDS compared to non-COVID-19 ARDS (90).

Thromboembolic complications might also be precipitated by underlying cardiovascular injury. For example, patients with co-existing ST-elevation MI and COVID-19 have significantly increased rates of thromboembolic complications, affecting multiple vessels and stents, thrombus grade post-percutaneous coronary intervention (115). Additionally, cardiac arrhythmias play an important role in the development of thromboembolic events, due in part to the shared underlying myocardial substrate (116). Cardiomyopathy, consisting of mechanical dysfunction, structural remodeling, and electrophysiological changes, is a common cause of both intracardiac thrombus and cardiac arrhythmogenic substrate formation (116). The presence of right-heart echodensity on transoesophageal and transthoracic echocardiography has been reported in COVID-19 patients (117–119). Interestingly, intracardiac thrombus coexisted with persistent tachycardia, global hypokinesis, left ventricular dysfunction, and right ventricular dilatation and reduced systolic function (117–119). Taken together, this indicates that thromboembolism in COVID-19 might be mediated via cardiac-specific pathologies.

At the mechanistic level, thromboembolic complications may arise due to activation of inflammation and hypoxia, platelet activation, endothelial dysfunction, and circulatory stasis in COVID-19. Inflammatory overdrive and hypoxia may induce abnormalities of coagulation, the third component of the Virchow triad. On necropsy, areas of diffuse and extensive inflammatory infiltrations have detectable thromboemboli and microemboli (120). Direct infection of immune cells with SARS-CoV led to activation of monocyte-macrophage differentiation, coagulation pathway upregulation, and increased cytokine production (121). SARS-CoV-2 might drive thromboembolic mechanisms by its utilization of the ACE-2 receptor, which is needed to clear Ang II from the circulation. Increased Ang II could, in turn, drive the release of vWF from endothelial cells and platelet activation via involvement of Na^+^/H^+^ exchanger (122). Finally, the presence of auto-antibodies, such as lupus anticoagulant, might drive activated coagulation pathways and thromboembolic risk (123).

Direct activation of platelets by SARS-CoV-2 is a likely pathway for the development of thromboembolism. Hottz et al. (124) reported platelet activation and formation of platelet-monocyte aggregates in patients with severe but not in mild COVID-19. Similar findings were observed when platelets from COVID-19 negative patients were treated with plasma from COVID-19 positive patients (124). Platelets from COVID-19 patients induces *ex vivo* expression of tissue factor (TF) in monocytes (124), indicating a likely reprogramming event during SARS-CoV-2 infection. Indeed, this hypothesis is supported by pre-publication evidence reporting the presence of SARS-CoV-2 RNA in platelets of COVID-19 patients, which were shown to be hyperactivated and aggregated at a lower threshold of *in vitro* thrombin stimulation (125). Platelets from COVID-19 degranulate, which correlates with reduced platelet factor 4 and serotonin levels, and release extracellular vesicles to participate in coagulation (125). Consequently, platelet reprogramming could facilitate the transmission of SARS-CoV-2 and promote thrombo-inflammation. Indeed, thrombo-inflammation mediated by distinct patterns of platelet and neutrophil activations, neutrophil-platelet aggregate formation, and neutrophil extracellular traps has been reported in COVID-19 pneumonia (126).

### Prophylaxis and Management of Thromboembolism in COVID-19

Given the high burden of comorbidities and mortality in patients with thromboembolic complications, proper and adequate anticoagulation is highly warranted. Current management of patients with severe COVID-19 includes subcutaneous low molecular weight heparin (LMWH), suspicion of venous thromboembolism in those with high D-dimer levels and rapid respiratory deterioration, and consideration of therapeutic anticoagulation in those in whom diagnostic testing is not possible and there is no apparent bleeding risk (127, 128). A retrospective series showed no mortality benefit with LMWH compared to non-users (129). However, in those with a high sepsis-induced coagulopathy score and markedly elevated D-dimer level, 28-day mortality was lower among users (129). There is also consideration of experimental interventions, such as plasma exchange or administration of anti-inflammatory drugs, in clinical trial settings.

Nevertheless, there are several unknowns with the management of thromboembolism and associated complications in COVID-19. For instance, will prophylactic as compared to therapeutic anticoagulation result in a better outcome in these patients? A prospective cohort recently demonstrated significant reduction in pro-coagulants 7 days after thromboprophylaxis (130). However, the study was very limited by sample size. In another study, patients on prophylactic anticoagulation had higher venous thromboembolism than the therapeutic anticoagulant arm, although the latter group had a higher overall incidence of thromboembolic events, including pulmonary embolism (131). It is envisaged that these issues will be answered in ongoing clinical trials, such as the COVID-19 HD, a randomized controlled trial comparing high-dose vs. low-dose LMWH (132).

## Summary

In addition to the known impact on the respiratory system, emerging evidence strongly implicates COVID-19 as a vascular disease. Patients with pre-existing cardiovascular conditions which are commonly characterized by endothelial dysfunction are particularly at risk of downstream complications and COVID-19-associated mortality. Endothelial cell dysfunction, inflammation, and damage are implicated as a consequence of the disease, which likely results in elevated ACS/AMI and thromboembolic risk in COVID-19 patients. Direct viral infection of the endothelium, as well as the surrounding pericytes, via the ACE2 receptor, are likely to be causative factors, as well as the deleterious effects of the supraphysiological increase of pro-inflammatory factors, the so called “cytokine storm.”

Clinicians and research scientists should consider monitoring the vascular effects of the disease to help identify and manage patients, which may highlight individuals at risk of cardiovascular complications. Despite therapeutic anticoagulation, COVID-19 patients remain at a high risk of both systemic and pulmonary venous thromboembolism. This highlights the need for, perhaps, a more aggressive anticoagulant therapy, and monitoring. Studies should explore the benefits of using D-dimer levels to guide treatment of thromboembolic complications. Further work is needed to determine how best to manage vascular inflammation in COVID-19 patients, which has the potential to significantly improve clinical outcomes in this pandemic.

## Author Contributions

All authors listed have made a substantial, direct and intellectual contribution to the work, and approved it for publication.

## Conflict of Interest

The authors declare that the research was conducted in the absence of any commercial or financial relationships that could be construed as a potential conflict of interest.
